# Distinct cortical encoding of acoustic and electrical cochlear stimulation

**DOI:** 10.1101/2025.08.01.668170

**Published:** 2025-08-01

**Authors:** Ariel Edward Hight, Michele N. Insanally, Julia K. Scarpa, Yew-Song Cheng, Michael Trumpis, Jonathan Viventi, Mario A. Svirsky, Robert C. Froemke

**Affiliations:** 1 Translational Neuroscience Institute, New York University Grossman School of Medicine, New York, NY 10016;; 2 Department of Otolaryngology-Head and Neck Surgery, New York University Grossman School of Medicine, New York, NY 10016;; 3 Department of Neuroscience, New York University Grossman School of Medicine, New York, NY 10016;; 4 Department of Otolaryngology, University of Pittsburgh School of Medicine, PA 15213; Department of Neurobiology, University of Pittsburgh School of Medicine, PA 15213; 5 Department of Biomedical Engineering, Duke University, Durham, NC, USA; 6 Department of Neurobiology, Duke University School of Medicine, Durham, North Carolina, USA; 7 Department of Neurosurgery, Duke University School of Medicine, Durham, North Carolina, USA; 8 Department of Neurology, Duke University School of Medicine, Durham, North Carolina, USA

## Abstract

Cochlear implants are neuroprosthetic devices that restore hearing and speech comprehension to profoundly deaf humans, and represent an exemplar application of biomedical engineering and research to clinical conditions. However, the utility of these devices in many subjects is limited, largely due to lack of information about how neural circuits respond to implant stimulation. Recently we showed that deafened rats can use cochlear implants to recognize sounds, and that this training refined the responses of single neurons in the primary auditory cortex. Here we asked how local populations of cortical neurons represent acute implant stimuli, using electrode arrays we developed for cortical surface recordings for micro-electrocorticography (μECoG), a form of intracranial electroencephalography (iEEG). We found that there was a limited tonotopic organization across recording sites, relative to a clearer tonotopic spatial representation in normal-hearing rats. Single-trial iEEG responses to acoustic inputs were more reliable than responses to cochlear implant stimulation, although stimulus identity could be successfully decoded in both cases. However, the spatio-temporal response profiles to acoustic vs cochlear implant stimulation were substantially different. Decoders trained on acoustic responses showed essentially zero information transfer when tested on electrical stimulation responses in the same animals after deafening and cochlear implant stimulation. Thus while acute cochlear implant stimulation might activate the auditory cortex in a cochleotopic manner, the dynamics of network activity are quite distinct, suggesting that pitch percepts from acoustic and electrical stimulation are fundamentally different.

## Introduction

Cochlear implants are neuroprosthetic devices that restore hearing and provides users with an ability to attain open-set speech perception without other aids such as lip-reading (Djourno and Eyries, 1957; Simmons et al., 1965; Michelson, 1971; House and Urban, 1973; Eddington et al., 1978; Hochmair and Hochmair-Desoyer, 1981; Wilson et al., 1991; Clark, 2006; Svirsky, 2017). Cochlear implants function by inserting a single and flexible shank of electrodes along the cochlear spiral (generally 12–22 channels in human subjects), to stimulate primary afferent neurons of the auditory system and bypass pathologies of deafness. Individual electrodes deliver trains of electrical pulses, providing spectral cues as a function of location along the topographical axis of the cochlear spiral, mimicking the encoding of sound in a functional and healthy cochlea. Over 1 million users have been implanted world-wide, making it the most widely adopted type of brain-computer interface, surpassing vagus nerve stimulators, deep-brain stimulation electrodes, and artificial retinas (Hays et al., 2013; Dawson et al., 2016; Ayton et al., 2020; Zeng, 2022; Johnson et al., 2024). Cochlear implants are thus the gold-standard for neuroprosthetic device use in terms of performance, safety, and ability to fine-tune or personalize the programming of each device to individual users.

Despite their wide adoption in human subjects over the last several decades, there is limited understanding of how these devices restore functionality and the sense of hearing to users. One long-standing question has been how the central auditory system responds to and interprets signals resulting from electrical stimulation of auditory nerve fibers. It is unknown to what degree emulating the patterns evoked by acoustic stimulation in normal hearing users is important for auditory perception. Many structures of the central auditory system are organized tonotopically, following the frequency alignment of the cochlear basilar membrane (Walzl and Woolsey, 1946; Merzenich et al., 1973; Polley et al., 2007). This topographic mapping might facilitate the encoding of auditory stimuli. While previous studies have demonstrated cochleotopic organization in auditory cortex of cochlear implant animals (Bierer and Middlebrooks, 2002; Johnson et al., 2016), a fundamental question remains unanswered: does electrical stimulation of specific cochlear locations elicit the same cortical representations and perceptual qualities as acoustic stimulation of corresponding frequencies? The spatiotemporal dynamics of these representations and how they relate to acoustic processing in normal hearing individuals remain unclear. Previous psychophysical studies in human implant users have provided indirect evidence for perceptual differences (McDermott, 2004) and similarities (Vermeire et al., 2013), but direct neural evidence comparing representations in the same subjects has been lacking. This question has profound implications for how we design and program cochlear implants, or more generally other braincomputer interface devices.

Studies in non-human animals are required to understand the neural basis of cochlear implant use and the relation to spatiotemporal representations in the auditory system. Responses to cochlear implant stimulation have been studied in a number of species generally in non-behaving animals under anesthesia (Klinke et al., 1999; Bierer and Middlebrooks, 2002; Middlebrooks and Bierer, 2002; Fallon et al., 2014; Johnson et al., 2016), but occasionally in awake or behaving animals (Vollmer and Beitel, 2011; Keating et al., 2013; Johnson et al., 2016). Over the last decade we have examined cochlear implant responses in deafened rats (King et al., 2016; Glennon et al., 2019), where we showed that animals can be trained to report the activation of specific implant channels. Behavioral training with the cochlear implant led to substantial plasticity within the deafened rat auditory cortex, modifying synaptic receptive fields and adjusting excitatory-inhibitory balance at the level of single neurons. Prior to training, the responses of single cells to implant stimulation were erratic and inhibition was mismatched relative to excitation. After training, inhibition became aligned with excitation across the array of implant electrode channels (Glennon et al., 2023). However, intracellular recording in vivo is infeasible in human subjects. Thus other approaches more amenable to clinical use are required, to measure neural coding of cochlear implant signals and enable translation of results from non-human studies to improve implant performance by human subjects.

Here we aimed to determine the spatiotemporal representations of implant channels beyond the responses of single neurons, but instead across a broader extent of rat auditory cortex. We take advantage of improvements in micro-electrocorticography (μECoG), a sub-type of intracranial electroencephalography (iEEG) that generally uses grid electrodes placed epi- or sub-durally over the cortical surface to measure local electrical activity (Insanally et al., 2016; Woods et al. 2018). iEEG recordings have high temporal precision, greatly improved signal-noise ratios, and better spatial resolution than conventional scalp EEG recordings (Abdi-Sargezeh et al., 2023; Janiukstyte et al., 2023). Signals reflect local electrical fields generated by aggregate neural activity in the regions adjacent to recording sites (Jasper and Penfield, 1949; Chang, 2015; Fukushima et al., 2015). iEEG has been used for studies of human brain organization particularly of speech and language centers, but these recordings are necessarily done in epileptic subjects to help locate seizure foci before surgical resection (Nourski et al., 2013; Mesgarani et al., 2014; Tang et al., 2017; Beynon et al., 2021; Norman-Haignere et al., 2022). Non-human animal studies remain essential for determining how sensory inputs are represented and processed in subjects without overt neurological conditions. To this end have designed and manufactured a novel and flexible 60-channel cortical surface iEEG array for adult rats, and validated that we can measure auditory responses and cortical map topography in normal hearing animals with these arrays (Insanally et al., 2016; Trumpis et al., 2017; Woods et al. 2018). Using these custom-fabricated iEEG grid electrodes, we now ask how auditory cortex responds to implant stimulation in untrained or trained deafened adult rats, and how different evoked signals and/or mesoscale tonotopic organization is in cochlear implant users vs normal-hearing animals.

## Materials and Methods

### Surgical procedure for iEEG recordings.

All animal procedures were performed in accordance with National Institutes of Health standards and were conducted under a protocol approved by the NYU Grossman School of Medicine Institutional Animal Care and Use Committee. We used a custom array consisting of 61 passive electrodes spaced 406 μm apart in an 8 × 8 grid, with three corner electrodes omitted and the fourth corner electrode intentionally unexposed to test encapsulation, reducing the number of active recorded sites to 60 (Insanally et al., 2016). iEEG grids were fabricated using a standard flex-PCB processing technique by Microconnex Flex Circuits (Snoqualmie, WA). All procedures and experiments were carried out in a sound-attenuation chamber.

A specific surgical protocol was developed for implantation of cortical surface iEEG in rats. Sprague-Dawley rats 4–6 months old were anesthetized with ketamine (40 mg kg−1, intramuscular injection) and dexmedetomidine (0.125 mg kg−1, intramuscular injection), or pentobarbital (50 mg kg−1, intraperitoneal injection). The head was secured in a custom head-holder that left the ears unobstructed. A longitudinal incision was made along the midline to expose the skull. Five bone screws were inserted into the skull around the point of entry of the electrode array to help anchor the dental cement (C & B Metabond Quick! Luting Cement). After reflecting the right temporalis muscle, a 5 mm × 5 mm craniotomy was made on the right temporal skull to expose the brain, and a sterilized iEEG array was epidurally placed over the left hemisphere core auditory cortex, located using vasculature landmarks. A thin silver wire was soldered between the headstage and the skull screws to be used as ground and reference.

### Surgical procedure unilateral cochlear implantation of the right ear.

Cochlear implantation procedures are similar to our past studies (King et al., 2016; Glennon et al., 2023). 8-channel animal cochlear implant arrays (HL08) were provided by Cochlear Americas. The array contained platinum-iridium band electrodes coated in silastic and connected to a nine-pin Nanonics connector (TE Connectivity, Berwyn, PA) with a single, additional extracochlear ball ground. The ipsilateral pinna was pulled forward and secured with a hemostat, the head rotated laterally, and the post-auricular junction between the ear canal and the sternocleidomastoid muscle (SCM) is identified as the initial incision site. An incision was made and the superficial fascia of the neck was dissected to identify the facial nerve i.e., cranial nerve (CN) VII. Minor bleeding was controlled using hemostatic epinephrine-soaked cotton pellets (Epidri pellets; Pascal International, Bellevue, WA), applied with light pressure. The SCM and posterior belly of the digastric muscle (PBD) were dissected from the tympanic bulla (TB) ventral and rostral to the trunk of CN VII. The TB was cleared of muscle and periosteum; the periosteum of the bulla was kept in normal saline and used later to seal the cochleostomy site. The drilling of the TB was begun ventrorostral to the trunk of CN VII with a 0.5 mm diamond burr and continued dorsally with care taken to avoid injuring CN VII, until the SA overlying the RW was fully visualized. Any remaining tissue or debris was removed with microforceps before the cochleostomy was performed.

Prior to performing the cochleostomy and inserting the array, the array lead and connector were secured. The post-auricular incision was expanded dorsally toward the skull. An area 4–5 mm in diameter was cleared and cleaned to expose the occipital skull, and the connector was attached perpendicular to the skull using C&B-Metabond (Parkell, Edgewood, NY) and bone screws. The lead to the electrode array was then sutured to the trapezius muscle, allowing enough lead to remain free to facilitate motion required for array insertion. The lead to the separate ground electrode was similarly secured into small muscle pockets in the trapezius. The cochleostomy site was identified ~0.5 mm directly below the lip of the RW in the basal turn of the cochlea, identified by both the stapedial artery and the cochlear promontory in the tympanic space. The site was gently drilled with a 0.1 mm diamond burr, and the array was inserted into the scala tympani without resistance using AOS forceps (Cochlear, Sydney, Australia) until all of the platinum-iridium contacts were within the scala tympani. The array occludes most, if not all, of the drill site, but to minimize postsurgical perilymphatic leak strips of periosteum taken from the bulla were placed around the implant to seal the site, followed by application of high-grade cyanoacrylate (Surgi-lock 2oc; Meridian Animal Health, Omaha, NE). The remaining lead was cemented into the bulla with C&B-Metabond (Parkell). Before closure, a small square of gelfoam with dexamethasone was left on the root of the facial nerve to prevent inflammation and heal any minor damage that may have occurred.

### Bilateral sensorineural hearing loss.

The deafening procedure was identical to the cochlear implantation procedure, with the exception of the array being removed before closure. Following both array and gelfoam removal, the cochleostomy site was closed with a trapezius muscle or periosteum graft, followed by 2-octyl cyanoacrylate (i.e., the array is removed from the cochlea). Both ears were deafened in this manner, but a functional array remained in the right ear for acute electrical stimulation.

### Behavioral training for tone and implant channel detection.

Four animals with iEEG recording electrodes were behaviorally trained to detect target tones. Rats were food restricted and trained on a self-initiated, auditory go/no-go task (Froemke et al., 2013; Martins and Froemke, 2015; King et al., 2016). Animals nosepoked in a designated port to initiate trials and are trained to nosepoke in a different port if the target tone was presented (4 or 22.6 kHz, any intensity) or withhold from nosepoking if a nontarget (foil) tone was presented (0.5–32 kHz excluding 4 kHz if 4 kHz was the target, or 8–45.3 kHz excluding 22.6 kHz if 22.6 kHz was the target, at 0.5–1 octave intervals and at any intensity). A sugar pellet reward was given for correct nosepokes within 2.5 s of target-tone presentation, whereas a 7 s timeout was given if the animal incorrectly nosepoked for foil tones. The four rats that achieved >70% target-tone hit rate and d’ ≥1.7 were included for further testing and implantation. A second subset of animals was behaviorally trained to detect cochlear implant channels; procedures were the same for tones except that a programmable clinical cochlear implant processor was used.

### Stimulus presentation for cortical sensory mapping in normal hearing rats.

Tone presentation was similar to our previous study of iEEG responses in normal-hearing rats (Insanally et al., 2016). Pure tones from 0.5–32 kHz (0.5–1.0 octave spacing) were generated using an auditory processor (TDT System III RZ6) and presented at a pseudorandom sequence at a rate of 1.25 Hz to the contralateral (right) ear using a calibrated free-field speaker (MF1 Multi-Field Magnetic Speaker, Tucker-Davis Technologies). Each tone was repeated 30 times at 70 dB SPL. The calibrated speaker exhibited <1% harmonic distortion and a flat output in the frequency range used.

### Stimulus presentation for cortical sensory mapping in cochlear implanted rats.

Electrical stimulation was delivered by an off-the-shelf Nucleus Freedom system speech processor (Cochlear) in which its transmitter coil drove a CI24RE implant emulator, where its output was connected to the implanted electrodes. The implant emulator is a standard clinical cochlear implant that is mounted in a plastic box with a DB-25 connector (Cochlear). We created a pigtail wire with a DB-25 connector and an Omnetics/Nanonics connector (Omnetics/TE Connectivity) to couple the emulator to the skull-anchored connector. The skull-anchored connector is also an Omnetics/Nanonics connector that is directly attached to the implanted array. For about half of tested animals (N=3), electrodes were activated through the microphone of the speech processor via tones presented to each individual electrode’s frequency allocation. The remaining cochlear implanted animals (N=4) were stimulated directly; the CI24RE implant emulator was driven by a Freedom system speech processor connected through the Freedom Programming Pod to a personal computer running the Custom Sound EP software (Cochlear). The Custom Sound EABR function was used (5 charge-balanced biphasic pulses, 25 μs/phase, 900 Hz stimulation frequency) to program and deliver stimuli to the implant.

### Cochlear implant programming.

Impedance and threshold measurements were obtained intraoperatively using Custom Sound EP (Cochlear) and were used for the initial programming of the sound processor. The ECAP thresholds were obtained and used to set the maximum stimulation level and the minimum stimulation level was set to 30 ‘clinical units’ below the maximum level, equivalent to 4.7 dB in the CI24RE implant emulator (Azadpour and McKay, 2012). All additional settings typically deployed in a clinical setting, such as ADRO, were turned off (Custom Sound, clinical programming software for Nucleus Freedom).

### Processing of cortical iEEG recordings.

All signal processing and analyses were performed using custom MATLAB scripts (MathWorks, MA). To determine whether trials were functional or contained artifacts, raw measurements were first filtered (2–150 Hz, 6^th^ order bandpass IIR filter) and then root mean squared power (rms) was computed for each channel. All trials with rms 20–300 μV were considered functional; power less than 20 μV corresponded with pre-amplifier saturation, while power greater than 300 μV consistently indicated intermittent corruption from non-neural sources. To then process functional recordings, we first used a notch filter to remove 60 Hz noise and 90, 120, 180 Hz harmonics. Recorded signals were then downsampled from 20 to 2 kHz using the decimate function (MATLAB), performing also the function of low-pass (anti-alias) filter and thereby rejecting any artifacts resulting from electrical stimulation delivered by cochlear implant electrodes.

Event-related potentials (ERPs) were extracted from raw downsampled recordings by further bandpass filtering (2–100 Hz) using a digital zero-order 6^th^ order Butterworth filter. Magnitudes of evoked ERP transients were measured by first rectifying measurements using the MATLAB abs() function and then subtracting the maximum of the baseline amplitude in the 50 ms pre-stimulus period (averaged over ±1 timestep) from the maximum of evoked amplitude in the 50 ms post-stimulus period (also averaged across three trials).

High gamma responses were extracted from raw downsampled recordings by further bandpass filtering (70–140 Hz) using a digital zero-order 6^rd^ order Butterworth filter. Next, signals were rectified and smoothed with a 20 ms sliding window. Resulting signals that exceeded 5 times the 90^th^ percentile were interpolated. Evoked high gamma magnitudes were extracted as for ERPs.

### Estimation of best frequency.

All analyses of tone-evoked responses were restricted to the range of 1.4–32 kHz tones. Best frequency was estimated by computing the center of mass of tone-evoked responses at 70 dB SPL. The response mass function was defined by the mean vector after projecting all responses on a circular domain where vector angles are represented by tone frequencies, to prevent biasing the center of mass towards the interior of the 1.4–32 kHz stimulus range.

### Principal component analysis (PCA).

Evoked responses were analyzed by including both temporal (i.e., *R* post-stimulus responses) and spatial (i.e., *S* responsive sites) variables. These measurements were concatenated across individual trials, *T*, to create a 2-dimensional *T* (rows) × *R*-*S* (columns) matrices. PCA was then performed and *R*-*S* vectors were compressed using singular value decomposition to obtain a reduced-rank approximation of the original matrix. PCA was also performed on variations of our data including only spatial *P* values (i.e., including only evoked magnitudes) or only temporal *R* values (i.e., averaging responses across all recording sites). The top 15 ranked projections were preserved for further analysis, reducing the datasets into a *T* (rows) × 15 (column) matrix.

### Tensor component analysis (TCA).

We applied a canonical polyadic (CP) decomposition to a 3-dimensional model, enabling us to treating spatial and temporal aspects of iEEG recordings across multiple trials as linear independent (orthogonal) dimensions (Kolda and Bader, 2009; Williams et al., 2018). Naive feature vectors were constructed by arranging all data into a three-dimensional matrix composed of length-*R* post-stimulus responses, *S* responsive sites, and *T* stimulus trials resulting in a *R* × *S* × *T* matrix. CP decomposition was performed (Bader et al., 2023) for 15 latent factors, the number 15 chosen to exceed the number of tone or implant stimuli and to parallel the data reduction performed by PCA. Then the resulting estimate of the *T* factors for each of the 15 components reduced our datasets into *T* (rows) × 15 (column) matrices.

### Linear discriminant analysis (LDA).

We trained a decoder for predicting stimulus identity from single trial recordings by developing a supervised linear classifier. Each decoder was retrained and retested 1,000 times to average decoder performance across randomizations for combinations of trials used for training and test sets. Specifically, each bootstrap replicate was randomly partitioned to include 13 trials per stimulus for each training set and the remaining trials being used to test the decoder. LDA was applied to estimate the stimulus-likelihood map of the PCA- or TCA-compressed feature space. Naive feature vectors from the training set were normalized and compressed according to the learned transformations, and the maximum likelihood stimulus (i.e., the identity of the tone frequency or active cochlear implant electrode) was estimated for each response. Absolute accuracy of the decoder was computed as the average accuracy over all stimuli was estimated by the total proportion of correct predictions. In addition to absolute accuracy, we also computed the mean of the error distances between the predicted and actual stimulus, i.e., octaves or number of electrodes.

## Results

### iEEG recordings in normal hearing and cochlear implanted rats.

**Figure 1.**
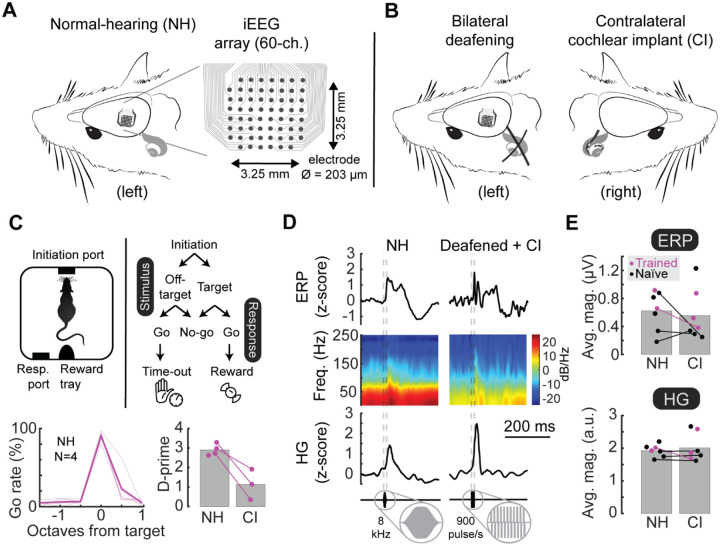
Auditory cortex iEEG responses to pure tone stimuli in normal-hearing and deafened cochlear implant rats. **A**, Schematic of 60-channel cortical surface electrode arrays, covering 3.25 × 3.25 mm on the surface of auditory cortex to record tone-evoked responses in normal-hearing (NH) trained rats. **B**, Some animals were bilaterally deafened and fitted with a unilateral cochlear implant (CI). **C**, Behavioral training of subset of animals on auditory go/no-go frequency recognition task. Animals were first trained when normal-hearing (NH, N=4, d’: 2.9±0.1) and then re-trained to respond to cochlear implant stimulation (CI, N=3, d’: 0.9±0.2). **D**, Examples of trial-averaged single-site iEEG responses (top, ERPs; middle, ERP spectrograms; bottom, HG). Clear transients were evoked by tones in normal-hearing animals (NH, left column) and in cochlear implant rats (CI, right column). **E**, iEEG response magnitude was similar between normal-hearing (NH, ERP amplitude: 1.9±0.1 μV; HG amplitude: 0.6±0.1 a.u.) and cochlear-implant rats (CI, ERP amplitude: 2.0±0.2 μV, p=0.43 compared to normal-hearing ERPs, Student’s paired two-tailed t-test; HG amplitude: 0.6±0.1 a.u., p=0.33 compared to normal-hearing HG).

We had two main goals: 1) to determine which features of auditory cortex iEEG signals were most informative about stimulus identities; and 2) to use iEEG recordings to assess cortical coding of acoustic vs electrical stimuli in normal-hearing (NH) vs bilaterally deafened + unilaterally cochlear implanted (CI) rats. Animals were first anesthetized and then a craniotomy was performed to expose the primary auditory cortex. We implanted a custom 60-channel iEEG recording array (Insanally et al., 2016; Trumpis et al., 2017) over primary auditory cortex. Seven rats were normal-hearing and presented with acoustic pure-tone stimuli (Fig 1A, ‘NH’). Seven rats were bilaterally deafened via cochleostomy before receiving a unilateral cochlear implant (Fig. 1B, ‘CI’).

A subset of these animals from each of the two groups (N=4 normal-hearing rats, N=3 cochlear implant rats) were trained on an auditory detection and recognition go/no-go task we have used previously, both for normal hearing (Froemke et al., 2013; Carcea et al., 2017; Insanally et al., 2024) and with cochlear implants (King et al., 2016; Glennon et al., 2023). Normal-hearing rats each achieved high levels of performance, whereas the cochlear implant rats had more variable performance (Fig. 1C; normal-hearing behavioral performance d’: 2.9±0.1, cochlear implant performance: 0.9±0.2).

We acquired and then compared acute iEEG recordings of acoustic tone-evoked and electrical cochlear-implant-evoked responses in normal-hearing and cochlear implant rats. Raw responses were down-sampled and lowpass filtered to eliminate electrical artifacts from cochlear implant stimuli, and bandpass filtered again either to reveal slowly varying event-related potentials (ERPs, bandpass range: 2–100 Hz) or to reveal higher frequency high gamma oscillations (HG, bandpass range: 70–140 Hz) (Fig. 1D). Trial-averaged evoked responses were time-locked to the stimulus onset. Evoked response magnitudes were not measurably different between NH and CI rats (Fig. 1E). We found that the behavioral training (denoted purple in all figures) had no measured effect on our analyses of sensory encoding compared to responses measured in untrained rats (Student’s unpaired two-tailed t-test, p>0.3 comparing normal hearing and cochlear implant signals in untrained vs trained rats).

### A1 encoding of tones and cochlear implant electrodes are spatially organized.

**Figure 2.**
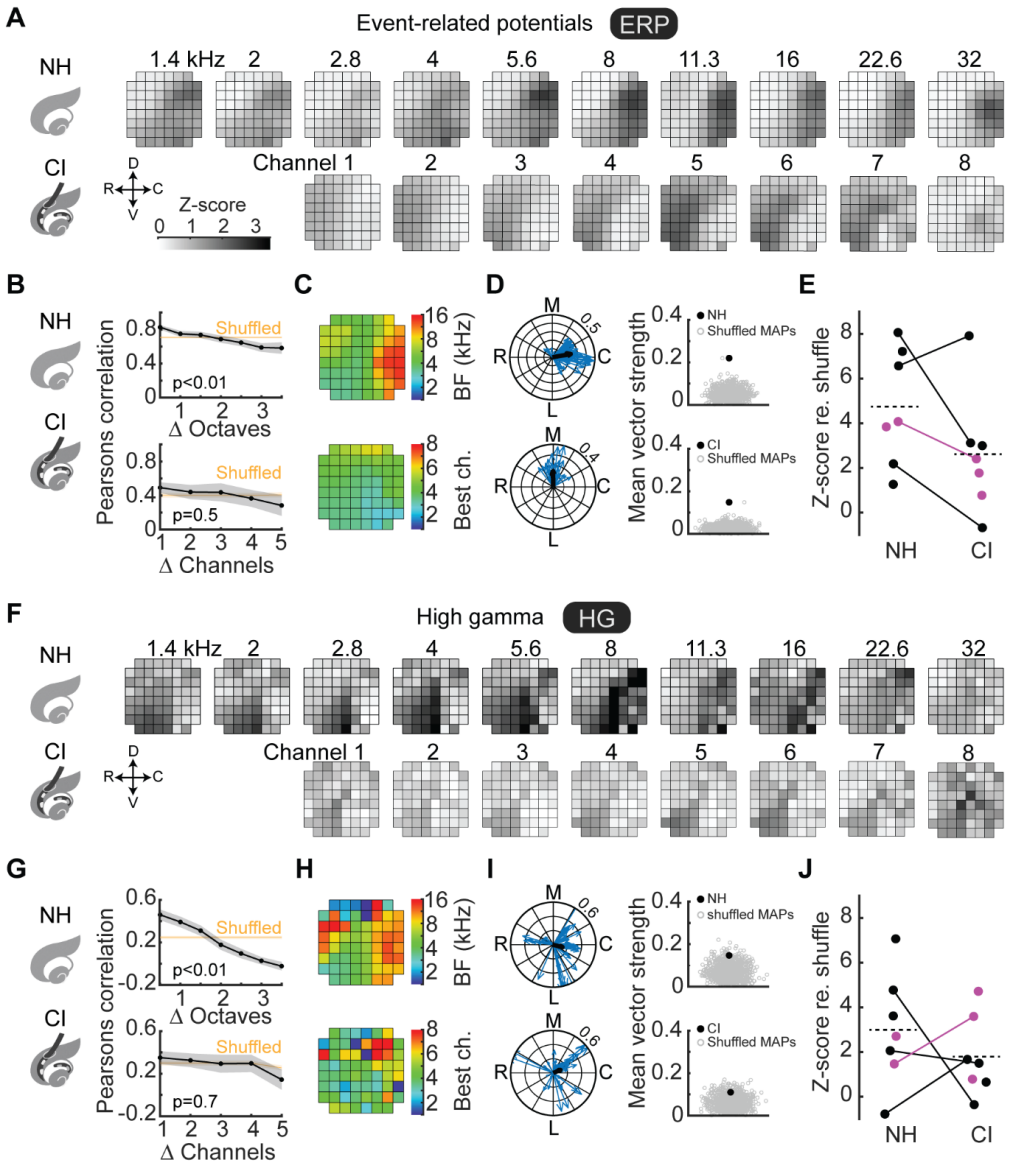
Tone-evoked and cochlear implant-evoked iEEG measurements are spatially organized. **A, F**, Trial-averaged tone-evoked event-related potentials (ERPs, panel **A**) and high gamma (HG, panel **F**) were spatially restricted to regions along the iEEG recording array; these active regions shifted as a function of stimulus. **B, G**, Spatial correlations of evoked response areas decreased monotonically as a function of increasing stimulus separation. Evoked iEEG responses as assessed by ERPs and HG were nonrandom for normal-hearing and implanted rats (p<10^−8^ compared to all shuffled responses), but were locally tonotopic in normal-hearing rats (p<10^−4^) but not implanted rats (ERPs: p=0.5, HG: p=0.7). **C, H**, Determining the preferred stimulus of spatially-evoked activity revealed smooth gradients shifting from high-to-low to high tone frequencies or cochlear implant channels (ERPs, panel **C**; HG, panel **H**). **D, I**, Local tonotopic gradients for each recording site plotted on a unit circle (blue) as a function of magnitude (strength of gradient) and angle (direction of gradient). The vectors across each recording site were averaged to produce an overall tonotopic vector (black) whose magnitude represents a metric of overall topography (ERPs, panel **D**; HG, panel **I**). The mean vector was plotted against the mean vector computed from n=1,000 shuffled maps (ERPs, panel **D**: normal-hearing z-score: 6.6, p<10^−10^; implanted z-score: 7.9, p<10^−14^; HG, panel **I**: normal-hearing z-score: 2.1, p=0.02; implanted z-score: 1.5, p=0.07). **E, J**, Z-scored magnitudes of mean tonotopic vectors across animals (ERPs, panel **E**: normal-hearing mean z-score: 4.7±1.0, implanted mean z-score: 2.6±1.0, p=0.22, Student’s paired two-tailed t-test; HG, panel **J**: normal-hearing mean z-score: 3.0±0.9, implanted mean z-score: 2.1±0.9, p=0.96).

iEEG recordings of tone-evoked responses are spatially restricted to areas across the recording array that appeared to shift as a function of tone frequency in normal-hearing animals (Fig. 2). Determining the best frequency at each recording site (i.e., the stimulus evoking the maximum response overall at that location) confirmed the general tonotopic organization of auditory cortex (Fig. 2A–E, ERPs; Fig. 2F–J, HG). Tone-evoked responses were similar in expected directions and gradients (Polley et al., 2007) indicating that iEEG measurements have the spatial resolution for testing whether evoked responses are cochleotopically organized.

We then asked whether individual cochlear implanted electrodes provide spatially restricted stimulation along the cochlear length and whether central auditory pathways would effectively preserve this cochleotopic encoding. We found that iEEG recordings of responses evoked by individual electrodes were spatially restricted to areas across the recording array (Fig. 2A,F) that appear to shift as a function of cochlear electrode location. To determine the degree of local and longer-range topography, we computed the spatial correlations as a function of stimulus separation, and compared the actual correlation coefficients to the distribution of responses when spatial locations were randomly shuffled. This analysis revealed a coarse topographic organization that was non-random (Fig. 2B,G; p<10^−8^ compared to all shuffled responses for ERPs and HG both for normal-hearing and implanted rats), but less sharply tonotopic relative to the tonotopic organization in normal-hearing animals assessed with pure tones (Fig. 2B, p<10^−4^ for ERPs and HG for normal-hearing rats; Fig. 2G, ERPs: p=0.5, HG: p=0.7 for implanted rats). Direct within-animal comparisons of normal-hearing vs implant maps (N=4) indicated that cochleotopic gradients were positioned in similar orientations and directions (Fig. 2C,H).

To quantify the degree and direction of potential spatial tonotopic or cochleotopic organization in the iEEG recordings, we computed the local gradients for each recording site (one example animal shown in Fig. 2D, ERPs; Fig. 2I, HG). Each gradient is a vector consisting of a magnitude and direction, and thus we then averaged these gradients from each recording site to get the overall cochleotopic vector, the magnitude of which is an index of spatial organization for a given animal. We shuffled the recording site location labels to generate putatively randomly organized maps (n=1000 random shuffles per animal), and compared the actual mean vector strength to the shuffled distributions. For this example animal, the ERP maps were significantly different from chance in terms of spatial organization (Fig. 2D, NH p=0.00003, CI p=0.0033), while this was more modest for the HG maps in this animal (Fig. 2I, NH p=0.02, CI p=0.07). This analysis revealed a similar kind of organization between ERP and HG maps of normal-hearing vs cochlear implant rats (Fig. 2E, NH mean z-score: 4.7±1.0, CI mean z-score: 2.6±1.0; Fig. 2J, NH mean z-score: 3.0±0.9, CI mean z-score: 2.1±0.9). Combined with results from Figure 2B,G, these analyses indicate that overall there was similar spatial organization to cortical iEEG responses in normal-hearing and cochlear implant animals, although the resolution of this topography was sharper for normal-hearing animals.

### Increased variability of trial-by-trial responses evoked by cochlear implant stimulation.

**Figure 3.**
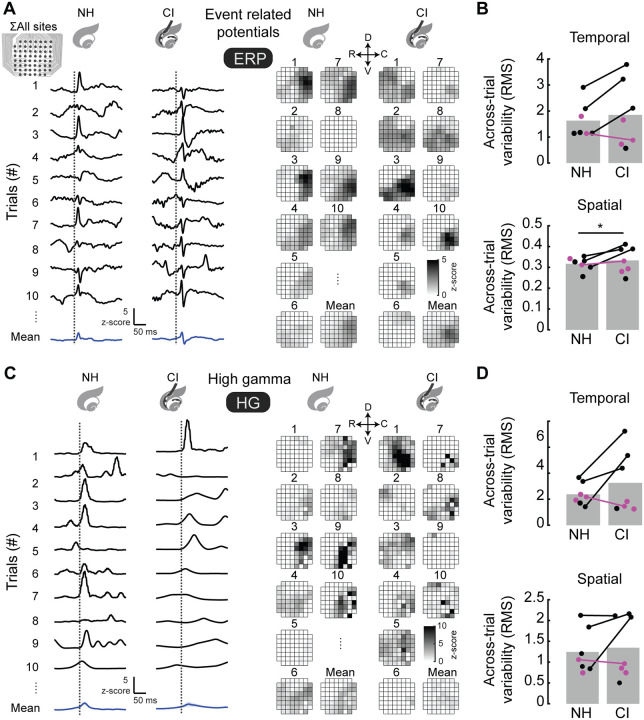
Trial-by-trial cochleotopic encoding is more variable in cochlear implant vs normal-hearing rats. **A, C**, Trial-by-trial evoked iEEG measurements in both normal-hearing and implanted rats revealed peaks across time (left) and space (right) (ERP, panel **A**; HG, panel **C**) **B, D**, Variability of iEEG measurements across trials (root mean square, rms) was consistently higher for cochlear implant-evoked compared to tone-evoked activity, for ERPs in panel **B** (top, ERP temporal NH mean rms: 1.6±0.3 over all animals, CI mean rms: 1.9±0.5, Student’s two-tailed paired t-test for animals monitored before and after deafening, p=0.12; bottom, ERP spatial NH mean rms: 0.32±0.01, CI mean rms: 0.33±0.02, p=0.05) and HG in panel **D** (top, HG temporal NH mean rms: 2.4±0.3 over all animals, CI mean rms: 3.3±0.9, p=0.18; bottom, HG spatial NH mean rms: 1.2±0.2, CI mean rms: 1.3±0.3, p=0.32).

The results described above for Figures 1 and 2 used trial-averaged responses to examine the spatio-temporal organization of iEEG responses. We noticed that the iEEG recordings provided satisfactory signal-to-noise ratios to measure evoked ERPs (Fig. 3A,B) and HG transients (Fig. 3C,D) on a single trial basis. Therefore, we next asked if there were differences in single-trial encoding for cochlear implant vs normal-hearing rats. We first averaged evoked responses across all recording sites to reduce measurement noise. We then computed variability of responses in the 50 ms period immediately following stimulus onset, and found that in this temporal domain, the within-animal trial-by-trial variability in evoked responses was similar for cochlear implant rats compared to normal-hearing rats irrespective of recording site (‘Temporal’; Fig. 3B, ERPs, Student’s paired t-test, p=0.12; Fig. 3D, HG, p=0.17).

Then, we returned to the evoked responses for each recording site and measured the trial-by-trial evoked response peak. Next, we computed the variability of spatial distributions of evoked responses and found larger within-animal trial-by-trial variability in evoked responses for CI compared to NH rats (‘Spatial’; Fig. 3B, ERPs, Student’s paired two-tailed t-test, p=0.05; Fig. 3D, HG, p=0.32). We conclude that similar to the trial-averaged responses in Figure 2, analysis of ERPs more so than HG signals could identify the increased spatial variability of evoked responses across the auditory cortex in cochlear implant animals relative to normal-hearing animals.

### Single trials encode stimulus identity in normal-hearing and cochlear implant rats.

**Figure 4.**
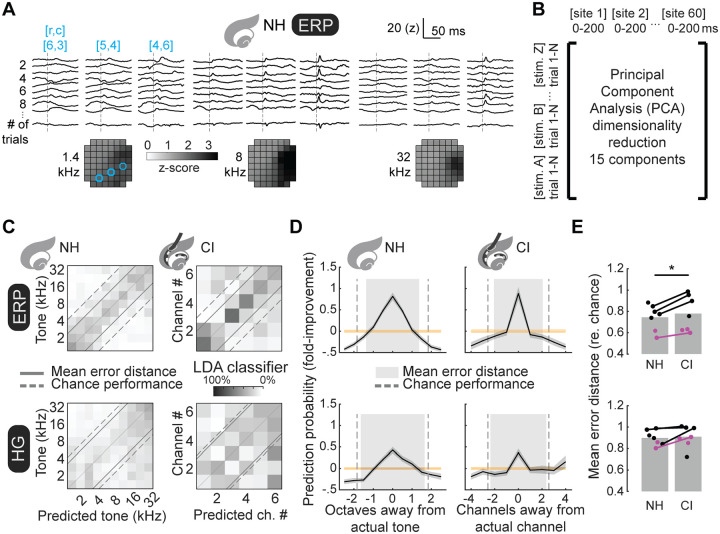
Trial-by-trial iEEG measurements encode stimulus identity. **A**, Trial-by-trial ERPs evoked by 1.4 kHz (left), 8 kHz (middle), and 32 kHz tones (right) are plotted for three recording sites (blue) reveal discernable evoked transients and spatially restricted patterns of evoked magnitudes (stimulus onsets depicted by dotted line). **B**, Trial-by-trial iEEG measurements concatenated such that columns represent recording sites (spatial) and post-stimulus sampling (temporal), rows by stimulus trials. Data then reorganized by PCA and reduced to the 15 components according to magnitude of explained variance. **C**, Classification matrices are plotted means across bootstrapped repeated (N=1,000) versions of LDA classifiers trained using 13 randomly selected trials for each stimulus, and classification predictions of single and remaining trials reveal significant prediction of stimulus identity (dashed line: chance-level error distance; solid lines: actual mean error distances). **D**, Stimulus prediction probabilities plotted across animals (black: mean, grey: s.e.m.) and as function of either octaves (normal-hearing) or channels (cochlear implant) from actual stimulus, reflecting a gradient in which adjacent stimuli share more encoded features than other stimuli. **E**, Decoder performance (mean error distance) across individual animals was somewhat worse for cochlear implant ERPs compared to tone-evoked ERPs (ERP mean error rate relative to chance for normal-hearing: 0.74±0.05, cochlear implant: 0.78±0.06; for animals assessed both first when normal-hearing and then after cochlear implantation p=0.02, Student’s two-tailed paired t-test), but not for HG (HG mean error rate relative to chance for normal-hearing: 0.90±0.03, cochlear implant: 0.91±0.04; for animals assessed both first when normal-hearing and then after cochlear implantation p=0.11).

Given that the trial-by-trial variability in evoked responses were higher for implant-evoked responses compared to responses in normal-hearing rats, we wondered if this would compromise the decoding of cochlear implant signals in some way. We next trained a classifier to determine if we could successfully decode stimulus identity from individual trials, and what differences there might be between decoding accuracy for normal-hearing vs cochlear implant rats. We reduced evoked responses into the top 15 principal components via PCA (Fig. 4A,B), and then trained an LDA decoder on 13 randomized trials of stimulus presentation to enable comparisons across animals that had different numbers of stimulus presentations (Insanally et al., 2016). Computed prediction probabilities were estimated by testing the decoder on the remaining trials. Selections of the 13 training and remaining test trials were randomized (N=1,000 trials), and final prediction probabilities were averaged across these repeats.

This approach successfully decoded tones and implant electrodes (Fig. 4C). Across animals, there were above-chance prediction of the correct stimulus from single trials, with higher errors when closer to the actual stimulus (Fig. 4D). Computing error distance mean across trials allowed us to compare decoding performance of normal-hearing vs implanted rats, as this combines absolute prediction probability and distribution of errors while normalizing to chance-level error distances, determined by the number of trained and tested stimuli. Decoders trained on responses from normal-hearing rats had better performance compared to responses from those animals after implantation (Fig. 4E; ERP mean error distance for normal-hearing rats: 0.74±0.05, cochlear implant rats: 0.78±0.06, p=0.02, Student’s paired two-tailed t-test; HG mean error distance for normal-hearing: 0.90±0.03, cochlear implant: 0.91±0.04, p=0.11).

### Single trial encoding is independently provided by both spatial and temporal cues.

**Figure 5.**
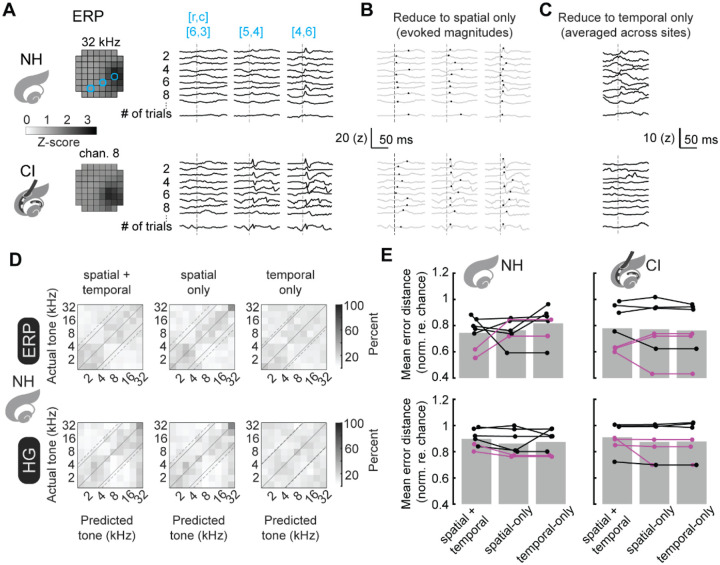
Trial-by-trial decoding from either spatial or temporal aspects of iEEG signals. **A**, Raw trial-by-trial ERPs for a single animal are plotted for 3 channels. **B-C**, Trial-by-trial iEEG measurements are reduced into either spatial only (**B**) or temporal only (**C**) by extracting either the magnitude of evoked activity or averaging across all recording sites. **D**, Re-training PCA-LDA decoders on spatial-only (middle) or temporal-only (right) does not abolish the encoding of stimulus identity. **E**, Decoder errors (mean error distance re. chance) across animals are plotted for decoders trained on spatial+temporal, spatial-only, and temporal-only iEEG measurements. Mean error distance for spatial+temporal ERPs, normal-hearing: 0.74±0.04, implant: 0.86±0.06; for spatial+temporal HG, normal-hearing: 0.90±0.03, implant: 0.97±0.04. Mean error distance for spatial-only ERPs, normal-hearing: 0.77±0.04, implant: 0.77±0.08; for HG, normal-hearing: 0.89±0.04, implant: 0.87±0.05. Mean error distance for temporal-only ERPs, normal-hearing: 0.82±0.05, implant: 0.81±0.07; for HG, normal-hearing: 0.87±0.04, implant: 0.93±0.05.

Which aspects of iEEG signals (spatial topography and/or temporal dynamics) contribute to stimulus decoding performance? We re-trained PCA-LDA decoders, but instead of using both spatial and temporal aspects of the full set of iEEG measurements (Fig. 5A), decoders were trained on the spatial-only (Fig. 5B) or temporal domain-only aspects (Fig. 5C) of evoked iEEG measurements. Decoders trained on the spatial or temporal domains only were essentially as good as predicting the stimulus as decoders trained on both aspects (Fig. 5D,E; mean error distance for spatial-only ERPs, normal-hearing: 0.77±0.04, implant: 0.77±0.08; for spatial-only HG, normal-hearing: 0.89±0.04, implant: 0.87±0.05; for temporal-only ERPs, normal-hearing: 0.82±0.05, implant: 0.81±0.07; for temporal-only HG, normal-hearing: 0.87±0.04, implant: 0.93±0.05; for spatial+temporal ERPs, normal-hearing: 0.74±0.04, implant: 0.86±0.06; for spatial+temporal HG, normal-hearing: 0.90±0.03, implant: 0.97±0.04). We conclude that both spatial and temporal aspects of iEEG measurements contribute to single-trial encoding of stimulus identity.

In some cases, we found that the performance of either spatial-only or temporal-only could be somewhat better than decoders trained on both spatial and temporal aspects of iEEG measurements. This is likely due to the noise inherent in iEEG signals, such that constraining PCA reductions to one aspect of the data (either spatial or temporal) helps to eliminate variance in individual recordings.

### Reducing evoked responses using TCA preserves encoding of stimulus identity.

**Figure 6.**
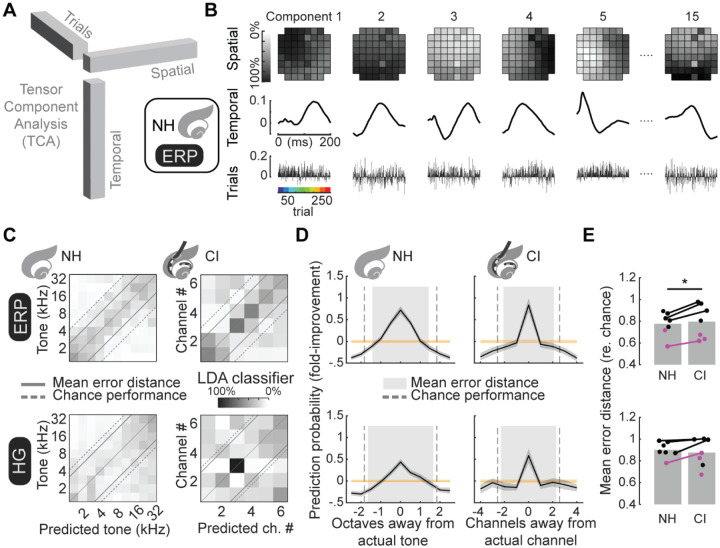
TCA-based decoding of single-trial iEEG measurements. **A**, Schematic of TCA with 3-dimensional tensors with orthogonal dimensions of spatial, temporal and trial factors. **B**, TCA-reduced iEEG measurements of stimulus-evoked ERPs in normal-hearing rats are plotted across 15 components of spatial (top row), temporal (middle row) and trial factors (bottom row). **C**, Classification matrices of mean decoder predictions across bootstrapped repeated (N=1,000) versions LDA classifiers trained using 13 randomly selected trials for each stimulus indicated that iEEG measurements encode stimulus identity from single stimulus presentations (dashed line: chance-level error distance; solid lines: actual mean error distances). **D**, Stimulus prediction probabilities are plotted across animals (black: mean, grey: std. error) and as a function of either octaves or channels in away from actual stimulus, normal-hearing and implanted rats. Correct prediction probabilities are high across all conditions and prediction errors coalescing of predictions toward actual stimuli (orange: mean and std. error of decoder performance on shuffled trials; grey: mean error distance, dashed line: chance level error distance). **E**, Decoder errors (mean error distance) across individual animals were slightly smaller for evoked iEEG measurements in normal-hearing compared to implanted rats (ERP mean error rate re. chance normal-hearing: 0.77±0.04, implanted: 0.80±0.06; HG mean error rate re. chance normal-hearing: 0.90±0.03, implanted: 0.88±0.05). Animals with both normal-hearing and implanted iEEG measurements only showed significant differences for ERP (top, Student’s paired t-test: p=0.01) but not HG (bottom, p=0.09).

One challenge with using PCA is that the ordinations of dimensionality reduction are unconstrained, because they are arbitrary linear combination of the original variables. A newer approach for dimensionality reduction called tensor component analysis (TCA) explicitly enables identification of which spatial, temporal, and single-trial factors best account for signal variability, and thus drive single-trial stimulus decoding (Williams et al., 2018). Here, we used TCA instead of PCA for LDA-based decoding, constraining data reduction dimensions on the full data set to the spatial, temporal, and to the trials of iEEG measurements (Fig. 6A). The resulting TCA-optimizations across 15 components reveal discernable patterns of spatial and temporal factors (Fig. 6B). We trained and tested a LDA decoder using only the trial factors, and found that single trial predictions accurately identified the stimulus (Fig. 6C–D). Decoders trained on responses from normal-hearing rats had better prediction performance compared to the responses from these same animals after cochlear implantation (Fig. 6E; ERP mean error distance for normal-hearing rats: 0.78±0.11, for cochlear implant rats: 0.80±0.15, p=0.01, Student’s paired two-tailed t-test; HG mean error distance for normal-hearing: 0.90±0.03, for cochlear implant: 0.88±0.05, p=0.1).

### Latent factors from unsupervised TCA revealed spatially-organized maps.

**Figure 7.**
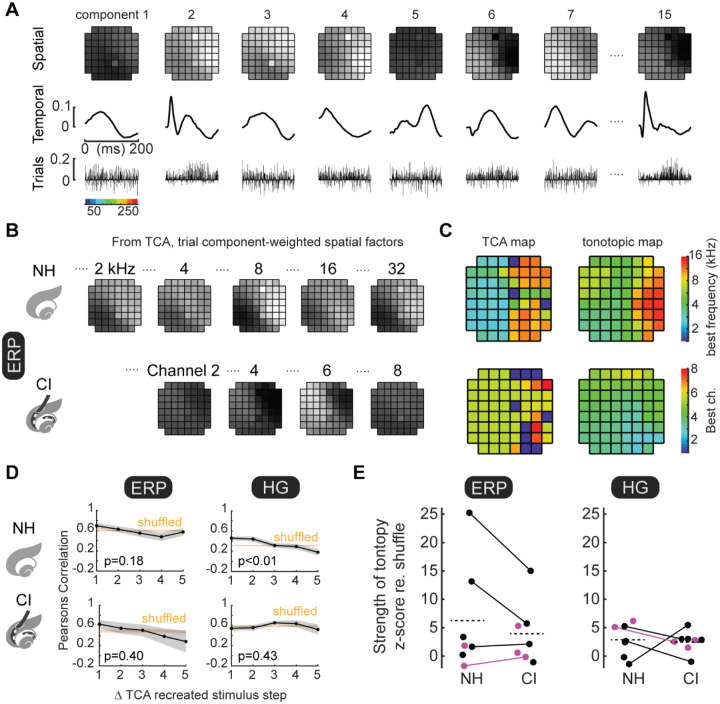
TCA data reductions reveal latent spatial factors are topographically organized. **A**, TCA-based analysis of evoked neural activity, divided into 15 unique components where each component is separated into orthogonal dimensions of spatial factors (top row), temporal factors (middle row) and trial factors (bottom row). **B**, Spatial maps for tone-evoked or electrode-evoked responses recreated from TCA reduced data. **C**, Reducing re-organized spatial factors revealed a TCA map (left) with topographical gradients in same location and direction as the tonotopic map reduced from raw measurements (right). **D**, Spatial correlations of evoked response areas decreased monotonically as a function of increasing stimulus separation. TCA-reduced models of evoked iEEG responses as assessed by ERPs and HG were nonrandom for normal-hearing and implanted rats (p<10^−8^ compared to all shuffled responses). TCA-reduced models were locally tonotopic only for HG in normal-hearing rats (normal-hearing ERPs: p=0.18, normal-hearing HG: p<10^−4^, cochlear implant ERPs: p=0.43, cochlear implant HG: p=0.40). **E**, Z-scored magnitudes of mean tonotopic vectors across animals were similar (ERPs, normal-hearing mean z-score: 6.2±3.6, cochlear implant mean z-score: 3.9±2.1, p=0.28, Student’s paired two-tailed t-test; HG, normal-hearing mean z-score: 2.9±1.1, implanted mean z-score: 2.5±0.7, p=0.88).

These analyses revealed that single-trial iEEG responses can be successfully decoded, and the TCA-based approach suggests that individual factors driving decoding might relate to specific spatiotemporal features inherent in the data. Specifically, we asked whether the TCA-reduced data contained organized spatial features, e.g., patterns of tonotopy. We next optimized TCA models of the original data (Fig. 7A) and then weighed the spatial factors by the strength of trial factors associated with each stimulus, resulting in spatial maps for each stimulus frequency for both normal-hearing and electrode for implants rats (Fig. 7B). The resulting reorganized spatial factors were further reduced to a preferred stimulus. Resulting TCA-maps exhibited tonotopic features (Fig. 7C), similar to tonotopic maps extracted from ERP or HG components of the iEEG responses. TCA-recreated spatial maps also exhibited coarse topographic organization that was non-random compared to shuffled distributions (Fig. 7D, p<10^−8^ compared to all shuffled responses for ERPs and HG both for normal-hearing and implanted rats). TCA models from normal-hearing HG iEEG measurements exhibited clear tonotopic organization whereas tonotopy in models from other measurements was less clear (Fig. 7D, normal-hearing rats ERPs: p=0.18, normal-hearing rats HG: p<10^−4^, cochlear implant rats ERPs: p=0.43, cochlear implant rats HG: p=0.40). As with analyses of Figure 2E,J, the magnitude of tonotopy computed from mean vector strengths was similar for ERP and HG measurements in normal-hearing vs cochlear implant rats (Fig 7E).

### iEEG measurements from implanted rats could not be decoded from models trained on normal-hearing data.

We asked what features might be shared vs distinct in the nature of cortical encoding of tones (in normal-hearing rats) and electrode channels (in cochlear implant rats). We hypothesized that this overlap would be incomplete, with substantial variability across individuals. This was based on a considerable number of previous studies finding that adaptation periods are needed before maximizing cochlear implant outcomes in both animals (Glennon et al., 2023) and humans (Holden et al., 2013; Cusumano et al., 2017; Glennon et al., 2020; Caswell-Midwinter et al., 2022), as well as our results described above showing that iEEG measurements were less sharply topographic and more variable in implanted rats compared to normal-hearing animals. Thus, here we asked whether a decoder trained on tone-evoked iEEG measurements from normal-hearing rats could interpret implant-evoked iEEG measurements on single trials.

**Figure 8.**
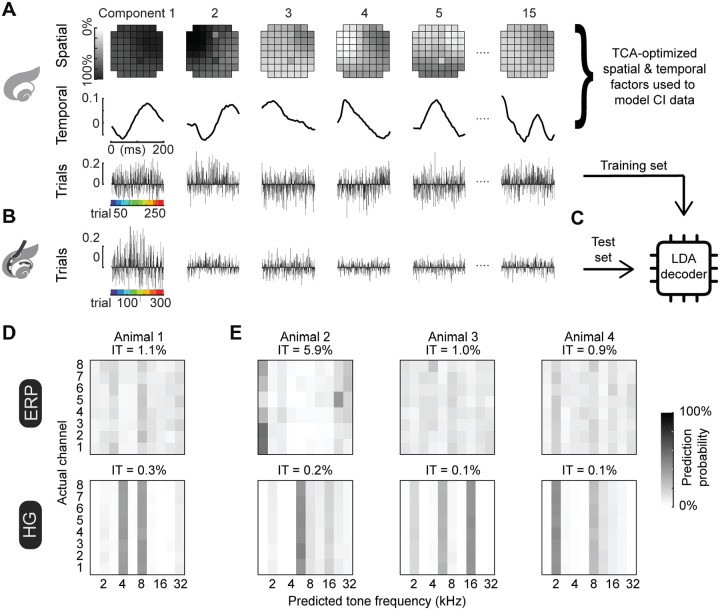
Lack of information transfer between acoustic and electrical stimulation representations in the same animals. **A**, Evoked iEEG measurements from a normal-hearing rat reduced using TCA. **B**, Evoked iEEG measurements in cochlear implant rat modeled using TCA, constraining the model with the spatial and temporal factors from the TCA model optimized on normal-hearing data, leaving only the trials as the optimizable variables. **C**, Linear discriminant analysis classifiers are trained on the trial factors extracted from the TCA-reduced models of evoked data in normal-hearing conditions and then used to predict stimulus identity from the trial factors from the TCA-reduced models of implant-evoked measurements. **D**, Classification matrices are plotted means of decoder predictions across bootstrapped repeated (N=1,000) versions of linear-discriminant analysis (LDA) classifiers reveal little information transfer (IT for animal 1; for ERPs: 1.1%, for HG: 0.3%). **E**, Classification matrices of normal-hearing trained and implant tested decoders reveal little-to-no information transfer in three other animals with iEEG measurements of both tone-evoked and implant-evoked responses (IT for animal 2, ERPs: 5.9%, HG: 0.2%; for animal 3, ERPs: 1.0%, HG: 0.1%; for animal 4, ERPs: 0.9%, HG: 0.1%).

Our measurements of iEEG activity in the same animals before and after deafening and implant fitting provide an opportunity to examine this directly, and determine how individually distinct the neural coding of sound is for the auditory cortex in both normal-hearing and cochlear implant conditions. TCA-generated models provide a reasonable approach to quantify the degree of information transfer from the normal-hearing to the implanted condition, because the dimensions of data reductions are predetermined (i.e., spatial and temporal), rather than unconstrained as with PCA models. We generated TCA models of tone-evoked iEEG measurements from normal-hearing rats (Fig. 8A). Next, we extracted only the spatial and temporal components of these normal-hearing TCA models to constrain models for implanted rats, leaving only the trial factors to be optimized (Fig. 8B).

Next, we compared these models across each animal by training an LDA decoder on the trial factors from tone-evoked TCA models and then testing the decoder on trial factors of the implant-evoked TCA models (Fig. 8C). From these resulting confusion matrices of prediction probabilities, we computed the mutual information between predictions of tone identity and actual implant channels to quantify how much tone predictions can be used to determine the actual identity of single channels, without imposing any hypotheses about how predicted tones map onto implant electrodes (e.g., tonotopy). We computed the pointwise mutual information for each combination of predicted tones and actual implant channels, which was then normalized by the joint probability. These resulting bits were combined across the entire confusion matrix to compute mutual information, and finally we converted this to a percentage of information transfer by dividing the experimentally measured mutual information by the total possible number of bits the matrix could provide.

We found the resulting information transfer to be low across all four animals where we measured both tone- and implant-evoked iEEG measurements (Fig. 8D,E; mean information transfer, ERPs: 2.2±1.1%; HG: 0.2±0.1%). The near-zero information transfer indicates that decoders trained on tone-evoked iEEG measurements do not make meaningful interpretations of implant-evoked iEEG measurements. We conclude that although iEEG responses can be meaningfully decoded from single-trial cortical responses (to either acoustic tones in normal-hearing animals or cochlear implant stimulation in deafened animals), the decoding algorithms and computations required are specific to the type of stimulus and do not immediately generalize between acoustic and electrical stimulation. This has implications for understanding the perceptual experiences of cochlea implant users e.g., pitch perception at acute implant stimulation. If the cortical representations were simply degraded or noisy versions of acoustic responses, we would expect partial information transfer. Instead, the mutual information analysis reveals that the acoustic response pattern does not inform the electrical stimulation response pattern. In turn, this suggests that the perceptual qualities (e.g., pitch) evoked by acoustic and electrical stimulation may be qualitatively different rather than simply degraded versions of each other.

## Discussion

Cochlear implants are the gold standard for success of brain-computer interfaces and neuroprosthetic devices to safely activate the human nervous system and effectively restore functional sensation (Hochmair et al., 2006; Wilson and Dorman, 2008; Clark, 2015; Svirsky, 2017; Glennon et al., 2020). While high levels of hearing and speech processing can be achieved by some users, outcomes remain highly variable in terms of learning rates and peak performance, especially in real-world conditions and even after controlling for age and durations of deafness (Blamey et al., 2013). Invariably, all users require an adaptation period; speech perception outcomes are initially poorer than later timepoints (Holden et al., 2013; Cusumano et al., 2017; Caswell-Midwinter et al., 2022; Stronks et al. 2025), indicating that both that acute encoding of cochlear implant stimulation by the central auditory system is not optimal and that neuroplastic processes are needed for maximizing outcomes. Current and future advances in engineering and implant programming algorithms might help improve outcomes further, but there is a general belief in the field that what is now required is an understanding of how the central nervous system responds to peripheral electrical stimulation (Kral et al., 2002; Kral and Eggermont, 2007; Wilson and Dorman, 2008; Moore and Shannon, 2009).

Our findings provide the first direct neural evidence that acoustic and electrical cochlear stimulation create fundamentally distinct cortical representations, with near-zero information transfer between modalities. This addresses a longstanding question in cochlear implant research: whether electrical stimulation of the cochlea can evoke the same percepts as acoustic stimulation, such as pitch and timing. The lack of transferable representations suggests that cochlear implant users may experience qualitatively different percepts that cannot be predicted from normal acoustic hearing. If so, this would challenge a foundational assumption of current cochlear implant design—that electrical stimulation should mimic the tonotopic patterns of acoustic hearing. Our data suggest that this biomimetic approach may be fundamentally limited because the cortical representations are non-overlapping.

Previous studies in non-human animals have established that stimulus identity can be encoded in A1 (Bierer and Middlebrooks, 2002; Middlebrooks and Bierer, 2002; Bierer and Middlebrooks, 2004). However, many of these studies have used invasive recording methods to examine single-neuron responses to implant activation. Invasive recordings are not feasible for human cochlear implant recipients, and thus decoding methods based on purely-invasive measures may be difficult to translate into human subjects for optimizing implant programming and/or training for use of the device. While our iEEG recordings are also invasive and under anesthesia, the principles of iEEG analysis for cochlear implant responses developed here might be more applicable to non-invasive EEG recordings or similar approaches more easily performed in humans. Grid electrodes allowed us to take simultaneous advantage both of temporal and spatial features in the data, some of which were more obvious in ERPs, others were more latent in HG signals or PCA/TCA factors.

Our results show that these features could provide accurate stimulus decoding even on single trials, indicating that: 1) use of these methods in translational or clinical settings could aid personalization and optimization of brain-computer interfaces, and 2) single-trial implant responses can be adequately processed by auditory cortex and presumably downstream regions related to perception and behavior. It has been unclear to what degree the patterns of tone-evoked and implant-evoked responses in normal-hearing vs deaf subjects are similar. We found that the encoding of cochlear implant electrodes was less reliable and identifiable than tone-evoked responses in normal-hearing animals. One caveat is that direct comparisons of evoked iEEG measurements between normal-hearing and implanted rats are challenging due to fundamental differences in the stimuli (e.g., the cochleotopic separation between stimuli such as the spacing between half-octaves vs spacing between successive CI electrodes), as well as the higher degree of inter-trial variability for implant responses (which is presumably not influenced by stimulus separation).

We also observed interesting differences in the information provided by ERPs vs HG signals. In terms of field potentials, the HG band is thought to most directly related to neuronal spike activity (Ray and Maunsell, 2011), which may account for why HG-evoked activity was more time-locked to stimuli and more spatially tuned compared to ERPs. Some single-channel HG measurements also had higher signal-to-noise levels than ERPs. A priori, it would seem that a decoder with access to both higher signal-to-noise measurements in the temporal and spatial domains should perform better at predicting stimulus identity. Instead, we found that decoding was more accurate with ERPs than HG. One possible explanation for poorer decoding performance with HG is that HG signals are more spatially tuned, leading to a smaller number of discernable channels with clear HG activity; accordingly, we found that across iEEG grid sites, there were more significant recording sites with ERPs than HG. HG responses also have higher trial-by-trial variability and are more temporally-constrained than ERPs, and thus the ERP might be more informative in terms of decoding performance.

Finally, we found that single-trial temporal factors could suffice to provide accurate decoding of implant channels. This suggests that preservation of clear cochleotopy in deaf subjects may not be a pre-requisite for successful implant use. Rather, it is likely that reductions in trial-by-trial and inter-neuronal variability might be more important as a mechanism for improving outcomes. This means that the neural responses to neuroprosthetic stimulation and use of brain-computer interfaces need not fully recapitulate responses to other modalities (e.g., acoustic stimulation) in space and time. Previously we showed that deafened rats could learn to use a cochlear implant to perform an auditory task, and that changes in cortical excitatory and inhibitory synapses related to implant use outcomes. In the current study, we did not track responses to implant use over time in relation to changes in perception and behavioral performance. Our results suggest that refinement particularly of inhibitory cortical responses might help increase inter-neuronal variance at the population level to increase spatial organization, while decreasing intra-trial variability at the single neuron level to enhance temporal organization and improve auditory perception.
